# Climate justice, mobility justice, and health inequities among racialized communities in Canada: a scoping review

**DOI:** 10.1186/s13690-026-01906-2

**Published:** 2026-03-29

**Authors:** Bukola Salami, Temiloluwa Fatokun, Sutherland Hedley Rezvani, Aloysius Nwabugo Maduforo, Gervin Ane Apatinga, Caitlin McClurg, Siu Ming Kwok, Aashima Rattan, Adebayo Majekolagbe, Anderson Assuah, Julie L. Drolet, Andreas Neef

**Affiliations:** 1 Department of Community Health Sciences, Cumming School of Medicine, University of Calgary, Calgary, AB T2N 4Z6 Canada; 2 Libraries and Cultural Resources, University of Calgary, Calgary, AB Canada; 3 School of Public Policy, University of Calgary, Calgary, AB Canada; 4 Centre For Newcomers, Calgary, AB Canada; 5 Faculty of Law, University of Alberta, Edmonton, AB Canada; 6 Aboriginal and Northern Studies Department, University College of the North, Manitoba, Canada; 7 Faculty of Social Work, Central and Northern Alberta Region (CNAR), University of Calgary, Calgary, AB Canada; 8 Arts, Education and Law Group, Griffith University, Brisbane, Australia

**Keywords:** Climate justice, Environmental racism, Mobility justice, Racialized health inequities, Canada, Indigenous health, Black health

## Abstract

**Background:**

Emerging evidence suggests that climate and mobility injustices disproportionately expose racialized and immigrant communities to substantial health challenges. Yet research on this issue remains limited in Canada, where these populations are rapidly growing and where such injustices are increasingly prevalent. This scoping review maps the existing literature at the intersection of climate justice, mobility justice, and health inequalities among racialized communities in Canada. Specifically, the review examines the intersections of climate justice, mobility justice, and health equity among racialized and immigrant populations in Canada to inform future research agendas.

**Methods:**

We conducted a scoping review of peer-reviewed literature published up to March 2025 using multiple bibliographic databases, including Medline, Embase, and Scopus, among other interdisciplinary sources with a combination of key search terms. Eligible studies were those based in Canada that examined climate-related health impacts on racialized populations and addressed at least one justice or mobility dimension. Data were extracted into Excel and synthesized thematically.

**Results:**

A total of 22 studies met the inclusion criteria, encompassing quantitative, qualitative, and mixed methods approaches. The majority of this research centered on Indigenous communities in northern and remote regions, with limited attention to Black populations and non-Indigenous immigrant groups. Reported health outcomes included food and water insecurity, infectious diseases, respiratory illnesses, mental health challenges, and injury risk. Climate hazards studied ranged from wildfires and flooding to permafrost thaw and extreme heat. Only a small number of studies incorporated disaggregated race-based data or considered mobility justice. While 14 studies employed conceptual frameworks, many did not include explicit justice-oriented analyses or integrate mobility dimensions.

**Conclusions:**

This review maps the emerging evidence on the intersection of climate justice, mobility justice, and health inequities in Canada, while providing critical insights for future research and interventions to promote mobility justice and health equity in the context of climate change. The findings suggest that although Indigenous-focused research has generated important insights, substantial gaps remain regarding non-Indigenous racialized populations and the explicit integration of mobility justice frameworks.

**Registration:**

No protocol was registered for this scoping review.

**Supplementary Information:**

The online version contains supplementary material available at 10.1186/s13690-026-01906-2.

**Table:** 

Text box 1. Contributions to the literature
• Synthesizes the existing Canadian evidence on the intersections of climate justice, mobility justice, and health inequities among racialized communities. • Identifies a strong concentration of research focused on Indigenous populations and highlights critical gaps in studies examining Black and other non-Indigenous racialized groups.
• Highlights the limited use of race-disaggregated data, intersectional approaches, and justice-oriented analytical frameworks in Canadian climate–health research. • Demonstrates how climate-related hazards and mobility constraints may compound health vulnerabilities through structural and systemic inequities. • Provides evidence-informed insights that may support more equitable public health policy, climate adaptation strategies, and mobility planning in Canada.

## Background

In recent years, the concepts of climate justice and mobility justice have attracted major global attention. Climate justice highlights how the impacts of climate change disproportionately affect low-income and Black, Indigenous, and other racialized communities [1, 2]. Climate justice frameworks also recognize how intersecting social structures such as class, gender, colonialism, and systemic racism shape differential exposure and vulnerability to climate risks [3]. Mobility justice, on the other hand, focuses on how inequities embedded within transportation systems shape access, health, and overall well-being, particularly for Indigenous and non-Indigenous racialized populations and may also influence people’s ability to respond to environmental hazards through evacuation, relocation, displacement, or access to essential services during climate-related emergencies [4].

It is well-established that climate change is a critical global challenge driven largely by anthropogenic activities such as greenhouse gas emissions, industrial processes, deforestation, and agricultural activities [5, 6]. Its consequences are profound, spanning economies, ecosystems, and societies. A growing concern is the increasing burden on human health, manifested through increased air pollution, the spread of infectious diseases, heightened psychological stress, malnutrition, and mortality from extreme climate-related events [6–8]. Although a global problem, growing evidence suggests that marginalized and vulnerable communities, who contribute the least to its causes, disproportionately bear its burdens [9–11]. This is particularly evident in high-income countries where systemic inequities and structural racism heighten the vulnerability of racialized groups, Indigenous peoples, immigrants and economically disadvantaged populations [12]. For instance, in Canada, Indigenous and racialized populations experience heightened vulnerability, shaped by systemic barriers in housing, healthcare, employment, and access to nutritious food [13–15]. Migrants and refugees face further challenges, including mental health concerns linked to displacement and limited access to essential services both before and after resettlement [16].

Inadequate mobility further exacerbates existing inequities. Reliable, accessible, and safe transportation, an essential determinant of health and well-being, remains a persistent barrier for Indigenous and racialized populations in urban and rural communities [17, 18]. Research suggests that limited mobility undermines physical and mental health as well as economic security, largely because it restricts access to healthcare, employment, education, nutritious food, and opportunities for social participation [17–19]. Moreover, infrastructure development aimed at improving mobility often disproportionately benefits affluent groups, leaving marginalized populations behind [17]. Unsurprisingly, studies also reveal that transit policing is more frequently enforced against people of colour [20, 21]. Furthermore, residential segregation compounds these disparities by limiting equitable access to public transportation, bike lanes, green spaces, and other neighbourhood amenities [22, 23], which may reinforce existing health inequities.

The intersection of the climate crisis with mobility injustice may further compound the challenges faced by racialized, Indigenous, and immigrant populations. Their lack of equitable access to resources, infrastructure, and opportunities further limits their capacity to cope with and build resilience against climate impacts. Moreover, when mobility options are scarce, people may be unable to evacuate, get to cooling centres in heat waves, or reach health facilities during climate-related illnesses. In this context, mobility can shape both exposure and vulnerability to climate-related health risks, as individuals with limited transportation or relocation options may face greater challenges responding to environmental hazards. This reality underscores the urgent need to embed climate justice and mobility justice into healthcare policies and broader policy frameworks to enable policymakers to more effectively address the intersecting health and mobility needs of equity deserving groups or communities and advance equity in the face of climate change.

While evidence is emerging on the connections between climate justice, mobility justice, and health inequalities, academic scholarship on this crucial topic remains limited and, in many cases, underexplored. In Canada, contributions are particularly sparse, despite the fact that racialized communities frequently experience climate and mobility injustices. Existing studies have largely focused on Indigenous communities, leaving the experiences of other racialized and immigrant groups understudied [24–26]. Yet Canada’s racialized and immigrant populations are growing, making it essential to identify and address the challenges they face, including those linked to climate and mobility inequities. Moreover, the Sustainable Development Goals (SDGs) emphasize “leaving no one behind,” which includes racialized and immigrant populations. Addressing these challenges is also consistent with Canada’s commitments to equity, diversity, inclusion, and accessibility. To better understand the scope and characteristics of existing research in this area, this study conducts a scoping review of literature at the intersection of climate justice, mobility justice, and health inequalities among racialized communities. The aim of this review is to map and synthesize the available empirical evidence and to identify key themes, conceptual approaches, and research gaps related to climate justice, mobility justice, and health inequities among racialized populations in Canada.

## Methods

This scoping review was designed in accordance with the five-stage methodological framework proposed by Arksey and O’Malley [27], with additional guidance from Levac et al. [28] to enhance analytic depth and methodological rigor. The reporting of this review follows the PRISMA-ScR (Preferred Reporting Items for Systematic Reviews and Meta-Analyses Extension for Scoping Reviews) reporting guideline to enhance transparency and reproducibility. A formal protocol for this scoping review was not registered; however, the review followed established methodological guidance for scoping reviews [27, 29]. The review aimed to map and synthesize empirical studies exploring the intersection of climate justice, mobility justice, and health outcomes among racialized populations in Canada.

### Stage 1: identifying the research question

The review was guided by the question: *What is the extent*,* range*,* and nature of empirical literature examining climate justice*,* mobility justice*,* and health outcomes among racialized communities in Canada?* This broad research question was designed to capture a wide variety of studies across disciplines, methodologies, and justice frameworks. The review also aimed to explore which theoretical frameworks or conceptual models were applied in this field and to identify gaps in population representation, conceptual development, and policy relevance.

#### Inclusion and exclusion criteria

In line with scoping review guidance from the Joanna Briggs Institute (JBI), the eligibility criteria were also conceptualized using the Population–Concept–Context (PCC) framework. Population: racialized populations in Canada, including but not limited to Black, Indigenous, Middle Eastern, South Asian, Southeast Asian, Caribbean, immigrant, refugee, or other racialized communities. Concept: climate- or environment-related exposures and their intersections with justice-related constructs such as climate justice, mobility justice, environmental justice, racism, inequity, or displacement. Context: studies conducted within the Canadian context that examined health outcomes or determinants of health.

##### Inclusion criteria

We included studies that addressed justice and mobility concepts, even if these terms were not explicitly mentioned, to capture implicit discussions of related themes. Studies were eligible for inclusion if they met all of the following criteria: (1) They explicitly addressed climate or environmental phenomena, including but not limited to climate change, disasters, pollution, extreme weather, flooding, or wildfires; (2) they focused on racialized or marginalised populations, such as Black, Indigenous, Middle Eastern, South Asian, Southeast Asian, Caribbean, immigrant, refugee, or racialized communities; (3) they engaged with justice-related constructs, including climate justice, mobility justice, environmental justice, racism, inequity, or displacement; (4) they were empirical in nature, using qualitative, quantitative, or mixed methods; (5) they were published in English or French; and (6) they were conducted within the Canadian context.

##### Exclusion criteria

(1) studies did not incorporate a justice-oriented framing, including climate justice, mobility justice, or environmental justice; (2) did not examine any form of health outcome, broadly defined to include physical, mental, environmental, or social determinants of health, was also excluded; (3) non-empirical studies, including opinion pieces and theoretical essays; (4) review articles, including literature, systematic, and scoping reviews; (5) They were conference abstracts, conference proceedings, or other non–peer-reviewed conference materials. (6) publications not in English or French; and (7) studies not conducted in Canada or lacking direct relevance to the Canadian context.

### Stage 2: identifying relevant studies

The development of a robust search strategy was undertaken in collaboration with a professional librarian at the University of Calgary. Drawing on her expertise, a comprehensive and interdisciplinary database search plan was developed to capture a wide range of empirical studies across disciplines including public health, social sciences, environmental studies, policy, and law. The final database search was conducted on 31 March 2025. No date restrictions were applied, allowing the search to capture all relevant literature available up to the time of the review.

Database searches included Ovid MEDLINE, Embase, PsycINFO, CINAHL, Global Health, Cochrane Central Register of Controlled Trials, Scopus, Web of Science, ProQuest, CAB Abstracts, Environment Complete, Canada Commons, Policy Commons, Social Work Abstracts, and SocINDEX. Reference chaining was also conducted to ensure a comprehensive search of relevant literature.

Search strategies incorporated both controlled vocabulary (e.g., MeSH, Emtree) and free-text keywords. Terms were grouped into three main concept clusters: (1) climate or environmental phenomena (e.g., “climate change,” “natural disasters,” “pollution,” “extreme weather,” “flooding,” “wildfires”), (2) racialized or marginalized populations (e.g., “Black,” “Indigenous,” “immigrant,” “refugee,” “BIPOC,” “racialized communities”), and (3) justice-related constructs (e.g., “climate justice,” “mobility justice,” “environmental justice,” “racism,” “inequity,” “displacement”). Boolean operators (AND, OR) and proximity operators (e.g., adj2, adj3) were tailored to each database’s syntax to ensure consistency and precision across platforms. The full search strategy for at least one database is provided in Supplementary Material 1 to support transparency and reproducibility.

### Stage 3: study selection

Search results were imported into Covidence systematic review software (Veritas Health Innovation, Melbourne, Australia), a review management platform. Duplicate records were removed automatically and manually verified. The screening process was conducted in two stages. During the title and abstract screening, two independent reviewers assessed each record for eligibility. Articles were retained for full-text review if they appeared to meet inclusion criteria or if their relevance was unclear. During the full-text screening stage, the same two reviewers assessed each article in depth, with a third reviewer consulted to resolve any disagreements.

**Fig. 1:**
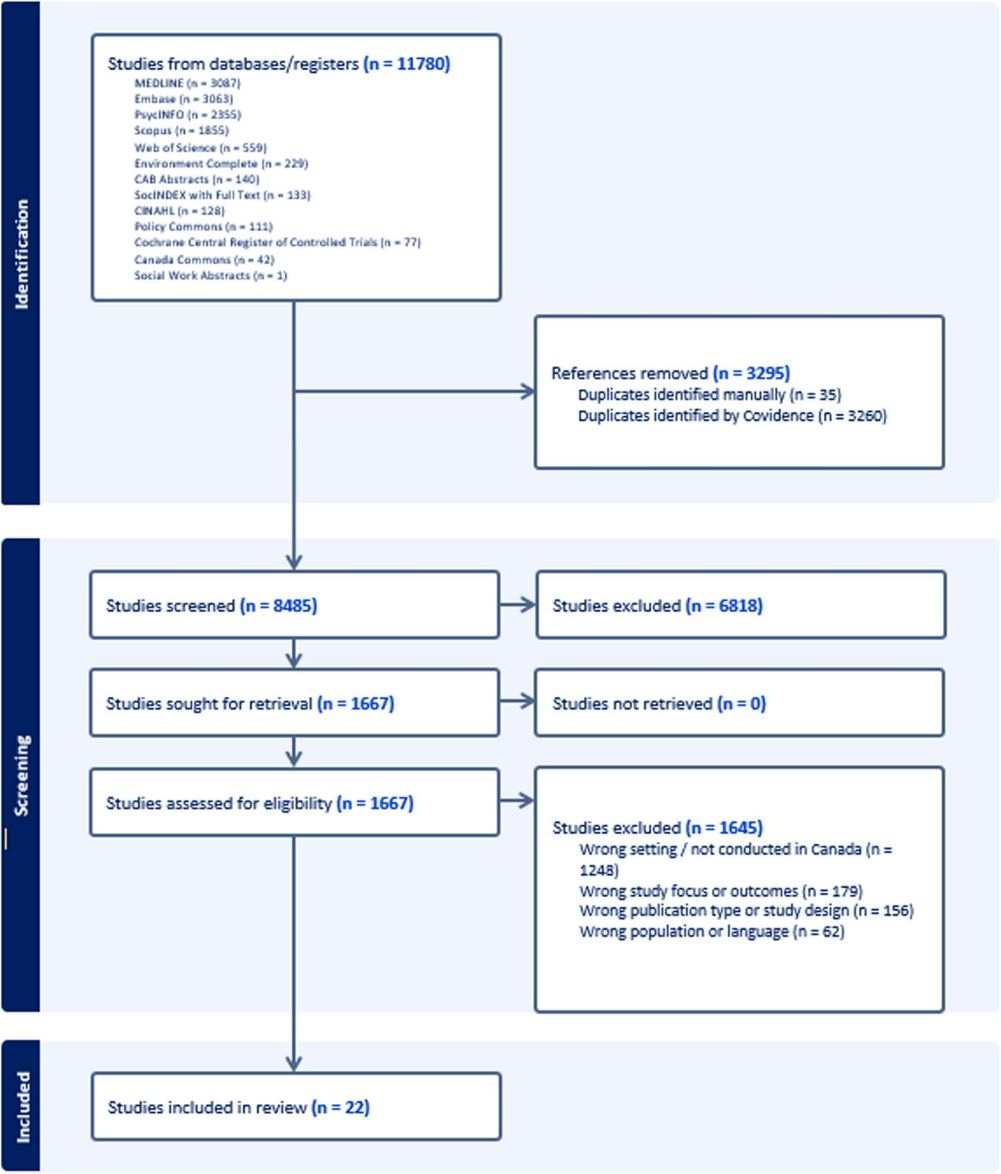
PRISMA-ScR flow diagram showing the study selection process, including records identified, screened, assessed for eligibility, and the final studies included in the review (n = 22). Adapted from the PRISMA Extension for Scoping Reviews (PRISMA-ScR) reporting guideline [29]. Legend: *MEDLINE* Medical Literature Analysis and Retrieval System Online, *Embase* Excerpta Medica Database, *PsycINFO* American Psychological Association database of psychological literature, *Scopus* Elsevier abstract and citation database of peer-reviewed literature, *Web of Science* Clarivate multidisciplinary citation database, *CINAHL* Cumulative Index to Nursing and Allied Health Literature, *CAB Abstracts* Centre for Agriculture and Biosciences International Abstracts database, *SocINDEX* Sociology Research Database, *Cochrane Central Register of Controlled Trials (CENTRAL)* Cochrane database of controlled trials, *Policy Commons* Global database of public policy documents, *Canada Commons* Canadian public policy database, *Covidence* Systematic review management software used for screening and data management, *PRISMA-ScR* Preferred Reporting Items for Systematic Reviews and Meta-Analyses Extension for Scoping Reviews

In total, 8,485 records were identified and screened. Following title and abstract screening, a subset was retrieved for full-text review. After applying the inclusion and exclusion criteria, 22 empirical studies met all eligibility criteria and were included in the final analysis. A PRISMA flow diagram was developed to illustrate the selection process (Fig. 1), including reasons for exclusion at the full-text stage.

### Stage 4: charting the data

A standardized data extraction form was created in Microsoft Excel (Microsoft Corporation) and pre-tested on a sample of five studies to ensure relevance and consistency. Data were extracted independently by two reviewers and verified for accuracy. The following fields were extracted: publication details (authors, year, journal), geographic location of the study, population characteristics (e.g., ethnicity, gender, immigration status), type of environmental exposure or climate hazard (e.g., heat waves, wildfires, flooding, pollution), health outcomes (e.g., mental health, chronic disease, access to care), mobility impacts (e.g., displacement, migration), methodological design, theoretical or justice frameworks used (e.g., environmental justice, Indigenous perspectives, intersectionality), and key findings.

In addition to empirical findings, the form also captured the conceptual framing, limitations noted by authors, policy recommendations, and whether population categories were aggregated or disaggregated. Particular attention was paid to whether the studies centered on marginalized racialized voices, and how systemic racism, colonialism, or other structural determinants were integrated into the analysis. Figure 1 (PRISMA flow diagram) is presented in the Results section.

### Stage 5: collating, summarizing, and reporting the results

Extracted data were analyzed using thematic synthesis. The analysis followed principles of thematic analysis as outlined by Braun and Clarke [30], involving familiarization with the data, coding, theme development, and synthesis. First, studies were grouped by population focus (e.g., Indigenous, Black, immigrant) and by type of environmental or climate hazard (e.g., slow-onset degradation, extreme weather, industrial pollution). Thematic coding was then conducted to identify major domains, including healthcare, food systems and agriculture, housing and infrastructure, education and knowledge transfer, cultural preservation, emergency preparedness, and climate-related migration. Within each domain, studies were further examined for how they articulated health impacts and whether they incorporated community strengths, culturally grounded knowledge, or resistance strategies.

Results were summarized in both narrative and tabular form to provide a clear and comprehensive overview of patterns in the literature. We paid particular attention to conceptual frameworks and theoretical depth, assessing the extent to which studies employed justice-based, anti-racist, or Indigenous frameworks. Studies that lacked theoretical framing were also noted, as this often corresponded with limited engagement with structural determinants of health.

Consistent with the objectives of a scoping review, a formal critical appraisal of the methodological quality of included studies was not conducted, as the purpose of this review was to map the breadth and characteristics of existing literature rather than evaluate study quality [27, 31].

Research gaps were identified based on population omissions (e.g., lack of studies on Black or non-Indigenous immigrant communities), underexplored health domains (e.g., mental health or disability), geographic clustering, and weak theoretical engagement. These findings informed the policy implications and future research directions outlined in the discussion section.

## Results

The study selection process is presented in Fig. 1. A total of 22 studies met the inclusion criteria, representing a small but growing body of research examining the intersection of climate change, mobility, and health among racialized populations in Canada. The included studies span multiple disciplines, including public health, environmental health, geography, and Indigenous studies, reflecting the interdisciplinary nature of climate–health scholarship. The studies were conducted across several regions of Canada, with a notable concentration in northern and Atlantic regions, where climate-related environmental changes are often more pronounced. Most studies focused on Indigenous communities living in rural, northern, or remote regions, while comparatively few examined urban populations or non-Indigenous racialized groups, highlighting a notable gap in the literature.

The included studies employed diverse methodological approaches. Eight studies used quantitative designs [25, 26, 32–37], seven used qualitative approaches [14, 38–43], and seven applied mixed-methods designs [13, 44–49]. Across the literature, research predominantly examined the experiences of Indigenous communities, with limited representation of Black or other non-Indigenous racialized populations. This distribution reflects the current focus of Canadian climate–health research on Indigenous contexts while indicating a clear need for expanded research involving other racialized communities.

Conceptual and theoretical frameworks were used in 12 of the 22 included studies, demonstrating varying levels of engagement with justice-oriented and Indigenous knowledge systems. Frameworks applied included environmental justice, Indigenous feminist theory, Two-Eyed Seeing, the Anishinaabe Medicine Wheel, and other Indigenous-informed approaches. The remaining ten studies did not explicitly specify a guiding theoretical framework, although some referenced justice-related concepts implicitly. This variation suggests that while justice-oriented perspectives are increasingly incorporated into climate–health research in Canada, their application remains inconsistent across studies.

Detailed characteristics of all included studies are presented in Supplementary Table 1, which summarizes publication details, geographic location, study design, population characteristics, conceptual frameworks, climate hazards examined, and key findings. This supplementary profile provides a comprehensive overview of the evidence base and supports transparency in the synthesis process.

Through thematic synthesis, six major domains emerged that map the current literature at the intersection of climate justice, mobility justice, and health among racialized populations in Canada:

- healthcare (*n* = 22).
- food systems and security (*n* = 8).
- environmental management and industrial development (*n* = 8).
- housing, education, Indigenous governance, and cultural preservation (*n* = 8).
- disaster and emergency response (*n* = 5).
- climate adaptation and migration health (*n* = 5).

These thematic domains highlight how climate-related environmental changes intersect with social and structural determinants of health. The findings collectively illustrate how climate hazards influence health outcomes through pathways such as food insecurity, environmental exposure, disruptions to healthcare access, and displacement-related stress. They also demonstrate how structural factors such as colonial legacies, geographic isolation, and uneven infrastructure shape vulnerability and adaptive capacity among racialized populations. Overall, the evidence base remains small and geographically concentrated, indicating that climate–health research involving racialized populations in Canada is still an emerging field.

### Healthcare

All 22 studies examined health in relation to climate change, with most addressing Indigenous populations. Fifteen studies focused on slow-onset climate hazards such as seasonal shifts, sea ice loss, and unpredictable weather, linking these changes to restricted mobility and reduced access to land-based activities. Tam et al. [33] was the only study that compared Indigenous and non-Indigenous populations, finding greater weather-related health impacts among urban Aboriginal populations, partly linked to acculturation stress.

Slow-onset hazards (*n* = 14) were repeatedly linked to restricted mobility and disrupted access to land-based activities, with contributing factors including: (1) seasonal shifts [14, 33, 36, 37, 42, 43, 46, 49]; (2) sea ice loss, thinning, and snowmelt [13, 14, 35, 36, 41, 42, 44–46, 48, 49]; and (3) unpredictable weather [13, 33, 37, 40, 41, 43–45, 49].

Mental and emotional health effects were frequently reported (*n* = 12), including: compounded impacts of climate change and intergenerational trauma [13, 14, 43]; stress and anxiety [13, 14, 33, 37, 40, 41, 43, 46, 48, 49]; depression [33, 37, 48]; and solastalgia [43, 46, 48] a term referring to emotional distress caused by environmental changes affecting one’s home environment. Ten studies also highlighted impacts on Indigenous sense of place, identity, and cultural wellbeing, emphasizing their deep connection to health and traditional land [13, 35, 37, 40, 41, 43, 45, 46, 48, 49].

Physical and nutritional health risks were discussed in twelve studies, including: death and injury from thinning sea ice [44, 46]; Lyme disease and cancer [43]; infectious gastrointestinal illness (IGI) [36]; diabetes and cardiovascular disease [48]; diet-related illnesses and malnutrition [35, 48]; respiratory or mold-related conditions [13]; digestive issues [40]; and hunger linked to socioeconomic stressors exacerbated by climate change [45, 49], with one study reporting hunger as a consequence of socioeconomic stressors aggravated by climate change [41]. Nine of these studies were based in Newfoundland and Labrador, where sea ice loss and changing landscapes were repeatedly tied to mental health challenges, injury risk, and food insecurity [13, 14, 35–37, 42, 44, 46, 48].

Out of the 22 reports reviewed, five examined slow-onset environmental degradation and exposure to pollutants [25, 26, 32, 34, 39]. Only two studies focused on non-Indigenous racialized populations [25, 26], both assessing the effects of air pollution (NO₂ and PM2.5) on health outcomes. Yu and Hu [26] examined differences in COVID-19 incidence, mortality, and hospitalization across socioeconomically disadvantaged neighborhoods, linking exposure patterns to racialized group membership. While the study suggested potential compounded risks from pollution and racialization, it did not directly quantify these combined effects. Stieb et al. [25] investigated air pollution-attributed mortality among older adults, finding higher risks in census tracts with more Indigenous and Black residents. However, they attributed much of this disparity to pre-existing structural inequities in baseline mortality rates, rather than pollutants alone.

Three other studies focused on Indigenous communities, analyzing a variety of pollution-related health impacts. These included: self-rated physical health declines [34], diabetes, obesity, and other pollution-linked illnesses [39], and cardiovascular disease, cancer risks, and nutritional concerns [32]. Mental, emotional, and spiritual health outcomes were also noted, with studies highlighting trauma from land loss and declines in cultural and spiritual wellbeing due to environmental degradation [34, 39].

Only one study examined a sudden-onset environmental hazard, reporting on a human-made flood that displaced Indigenous communities near Winnipeg [38]. The study documented high stress, anxiety, exacerbated chronic illnesses (e.g., diabetes, hypertension), and fatalities, including suicide, linked to the disaster and forced displacement. Finally, one study [47] raised concerns about environmental exposures potentially contributing to inflammatory bowel disease (IBD) among South Asian children, highlighting the limited number of studies examining immigrant populations in the Canadian climate-health literature.

### Food systems and security

Seven studies in this review examined how climate change affects food systems and agricultural practices among Indigenous communities in Canada. Across these studies, a consistent pattern emerged linking environmental change to disruptions in traditional food systems, with implications for nutritional health, cultural continuity, and community resilience. Warming temperatures, shifting seasonal patterns, and declining sea ice were repeatedly reported as factors that disrupted traditional harvesting activities and reduced access to culturally important foods.

Ford and Beaumier [49] examined food insecurity in Inuit communities, highlighting that unpredictable weather and ice conditions reduced access to traditional hunting grounds, while the high cost and limited availability of market foods compounded nutritional vulnerabilities. Several studies reported that reliance on market foods was associated with reduced intake of key nutrients, including iron, calcium, vitamin D, and omega-3 fatty acids, which are traditionally obtained through country foods. Similarly, Rosol et al. [35] and Wesche and Chan [45] found that climate-driven disruptions to wildlife populations (caribou, seal, whale, birds, and fish) diminished food availability, leading to greater dependence on market foods. In Wesche and Chan’s study, muskox and moose availability offered only partial substitution, which did not fully compensate for the nutritional and cultural losses associated with declining access to traditional foods.

Beyond nutritional impacts, several studies highlighted the cultural and psychosocial significance of traditional food systems. Cunsolo Willox et al. [48] and Harper et al. [13] described how unstable ice, warmer temperatures, and altered weather patterns have eroded access to traditional land-based food systems. These disruptions affected not only dietary patterns but also opportunities for intergenerational knowledge transfer and land-based cultural practices. This disruption not only compromised nutritional intake but also contributed to emotional distress and cultural loss, particularly among youth, due to declining opportunities for intergenerational knowledge transfer Cunsolo Willox et al. [48] introduced the concept of “solastalgia,” capturing the psychological pain of environmental degradation and loss of homeland. Harper et al. [13] highlighted that supply chain interruptions further compounded nutritional deficiencies and mental health challenges. Rosol et al. [35] examined food security programming in Nunavut, finding that while local initiatives exist, they often fail to address systemic drivers of food insecurity. The study emphasized the lack of justice-based frameworks and Indigenous self-determination in designing and governing food programs.

Calder et al. [32] also noted that colonial histories and environmental degradation intersect to limit access to traditional food sources. Their findings revealed a growing reliance on processed foods and highlighted how gendered constraints further limit harvesting activities. The study stressed the importance of protecting traditional food webs from contamination and promoting traditional foods as central to Indigenous identity and health. Chapola et al. [40] was the only study focused on agricultural practices, documenting the loss of medicinal plants and localized agricultural knowledge due to environmental change. These findings suggest that climate change may also threaten culturally embedded food and health practices tied to specific ecological conditions.

Despite the range of environmental changes described, none of the seven studies examined food insecurity or agricultural impacts among Black or other non-Indigenous racialized populations in Canada. Moreover, no studies employed an explicit food justice framework or analyzed structural determinants such as systemic racism or urban inequities. This absence of intersectional analysis across race, gender, and geography highlights an important gap in the Canadian literature on climate change, food systems, and health equity.

### Environmental management and industrial development

Eight studies examined how environmental management systems, water insecurity, and industrial development intersect with climate-related health impacts, particularly for Indigenous communities in Canada. Across these studies, a recurring pattern emerged linking environmental governance failures, industrial activity, and climate-related environmental change to adverse health outcomes and disruptions to community wellbeing. These studies highlighted systemic under-regulation of industrial projects, inadequate environmental monitoring, and climate-related disruptions to water access as major concerns.

Calder et al. [32] assessed potential health risks from hydroelectric dam development in Nunatsiavut, projecting increased methylmercury (MeHg) concentrations in aquatic food webs central to traditional diets. Similarly, Lewis et al. [34] investigated chronic industrial pollution from a pulp and paper mill affecting Pictou Landing First Nation, emphasizing that contamination of sacred water sites requires culturally grounded, community-driven monitoring and Indigenous data sovereignty to mitigate harms. Together, these studies illustrate how industrial development can compromise environmental health and traditional food systems when monitoring and governance mechanisms are insufficient. Both studies framed these impacts as clear examples of environmental racism, though they did not explicitly apply a climate justice lens.

Water insecurity was another recurring issue. Galway et al. [43] found that climate change exacerbated challenges to safe drinking water access in Indigenous communities in Ontario, where aging infrastructure and underinvestment increased vulnerability to changing precipitation and temperature patterns. Harper et al. [36] linked extreme weather events to increased cases of gastrointestinal illness in Nunatsiavut, recommending community-based water monitoring as an adaptive public health strategy. Middleton et al. [37] reported similar findings, highlighting that climate variability compromised water and wastewater infrastructure, with limited adaptation planning to address these risks. Collectively, these findings suggest that climate-related environmental changes interact with longstanding infrastructure inequities to heighten health risks in many Indigenous communities.

Tobias and Richmond [39] analyzed industrial development using a political ecology of health framework, showing how dams, resource extraction, and other projects disrupted water access, undermined self-determination, and caused environmental dispossession. Their study highlighted that forced geographic and political displacement eroded land-based knowledge, cultural continuity, and overall well-being. Similarly, Cunsolo Willox et al. [48] documented Inuit experiences of disrupted water access and environmental degradation, emphasizing cumulative psychosocial and cultural harms linked to these changes.

Stieb et al. [25] conducted a spatial analysis of air pollution-attributable mortality across seven major Canadian cities. They found that census tracts with higher proportions of Indigenous and Black residents experienced disproportionate long-term exposure to nitrogen dioxide (NO₂) and fine particulate matter (PM2.5), leading to elevated mortality rates. These disparities were tied to urban planning inequalities, residential segregation, and intersecting socioeconomic vulnerabilities. This study provides one of the few quantitative examinations of environmental exposure among non-Indigenous racialized populations in Canada within the climate-health literature. While this study underscores the need for equity-oriented environmental policies, it did not fully address mobility or displacement impacts.

Overall, the eight studies in this theme primarily focused on Indigenous populations [32, 34, 36, 37, 39, 43, 48], with only Stieb et al. [25] addressing other racialized groups. Few studies explicitly incorporated climate justice or mobility justice frameworks, and none examined the combined effects of environmental degradation, displacement, and mobility constraints. This points to a critical research gap on how environmental management failures, industrial pollution, and water insecurity affect Black and other racialized populations in Canada, despite well-documented evidence of environmental racism and inequities in these communities.

### Housing, education, indigenous governance and cultural safeguarding

Eight studies examined the intersecting domains of housing, education, Indigenous governance, and cultural preservation, showing that climate-related injustices are shaped not only by environmental degradation but also by housing insecurity, threats to cultural continuity, and barriers to community-based governance and knowledge transmission. Across these studies, governance, housing conditions, and cultural continuity emerged as interconnected determinants of climate resilience and health among Indigenous communities.

Six studies foregrounded Indigenous governance as central to climate justice, positioning self-determination, land stewardship, and cultural continuity as foundational to climate adaptation [14, 34, 38, 42, 43, 48]. These works highlighted how colonialism has undermined Indigenous identity through the displacement and degradation of lands, and emphasized governance frameworks rooted in Indigenous worldviews, such as Two-Eyed Seeing (Etuaptmumk), the Anishinaabe Medicine Wheel, and Mi’kmaw theoretical approaches [34, 43]. These frameworks supported culturally grounded risk monitoring and adaptation strategies, linking community resilience to continued access to land for mobility, cultural practices, and knowledge transmission despite climate disruptions [42]. Ballard et al. [38] underscored how jurisdictional fragmentation and chronic underfunding in housing infrastructure limit First Nations’ capacity for climate adaptation, framing governance both as a resilience strategy and as a right constrained by settler-colonial governance systems.

Housing conditions and insecurity emerged as an essential climate justice concerns in two studies. Ballard et al. [38] reported that inadequate housing, rooted in policy neglect and underfunding, left First Nations communities highly vulnerable to climate-related impacts. Chapola et al. [40] added that climate change has reduced availability of medicinal plants and opportunities for land-based practices, further compounding housing and livelihood vulnerabilities for Inuit communities in cold-climate regions.

Two studies examined education and cultural continuity as pathways for resilience and intergenerational knowledge transfer. Harper et al. [13] explored how culturally relevant education and land-based learning in Labrador contributed to youth engagement in climate adaptation, while acknowledging that colonial legacies have restricted opportunities for knowledge transmission. Cunsolo Willox et al. [48] described how climate change disrupted hunting, food sharing, and time spent on the land, framing these losses through the concept of solastalgia, defined as emotional distress caused by environmental changes affecting one’s home environment and sense of belonging. Both studies reinforced the need to protect and strengthen culturally grounded education systems as part of climate resilience strategies.

Several studies also employed conceptual frameworks to guide analysis. Galway et al. [43] and Lewis et al. [34] applied Two-Eyed Seeing alongside Indigenous knowledge systems, Middleton et al. [42] used a resurgence framework to analyze displacement and emergency management, Ballard et al. [38] employed a coloniality and environmental justice lens, Cunsolo Willox et al. [48] drew on an EcoHealth framework, and Chapola et al. [40] applied Climate/Environmental Justice and Indigenous Feminist frameworks to examine intersectional climate injustices.

Notably, none of the studies in this theme addressed the experiences or needs of Black or other racialized non-Indigenous communities in Canada. This absence highlights an important gap in climate justice research, as these populations may also encounter systemic barriers to housing, culturally relevant education, and environmental decision-making shaped by structural racism and colonial legacies.

### Emergency response and preparedness

Five of the twenty-two studies addressed disaster and emergency response and preparedness, all situated within Indigenous communities [13, 38, 40, 44, 46]. Collectively, these studies examined both slow-onset and sudden-onset environmental catastrophes, demonstrating how climate-related hazards place considerable strain on emergency systems while exposing longstanding structural inequities affecting Indigenous communities.

One study documented the mental, emotional, and physical strain resulting from a human-made flood that was deliberately rerouted to protect Winnipeg at the expense of 17 First Nations communities [38]. The flooding displaced communities, leading to unstable housing and experiences of racism in host cities. The authors argued that these impacts were rooted in structural neglect and called for policy changes such as involving Elders in advisory roles, creating culturally appropriate disaster plans, offering mediation services to address internal conflicts, and tailoring communication strategies to the needs of First Nations communities.

Similar issues were noted in research linking climate change-driven thinning sea ice with search and rescue (SAR) operations in Inuit communities [44]. Although trends in SAR incidents were inconsistent, interviews revealed substantial underreporting of incidents, pointing to gaps in systematic injury surveillance that hinder long-term planning. Other studies [13, 40, 46] described the compounding effects of severe weather on physical and mental health, including injury, anxiety, and illness. Harper et al. [13] emphasized that unpredictable weather patterns disrupt multiple determinants of health, calling for the integration of EcoHealth principles, Indigenous representation in climate-health planning, and enhanced community engagement.

Durkalec et al. [46] further argued that colonial policies, including the Indian Act, have disrupted Indigenous land-use patterns, making land-based strategies for adaptation less effective. The authors emphasized the importance of understanding the cultural significance of place when designing emergency management and climate adaptation strategies. Chapola et al. [40] underscored the lack of support in key areas such as mental health services, sanitation, housing, and land rights, particularly for Indigenous women, and called for strategies that strengthen community cohesion, expand mental health infrastructure, and amplify Indigenous women’s voices in emergency planning.

### Climate adaptation and migration health

Five studies explored the relationship between climate change, migration or mobility, and the health of racialized populations, including adaptation strategies to mitigate climate- and environmentally induced stressors [14, 36, 38, 46, 47]. Across these studies, climate-related environmental change was linked to mobility constraints, displacement, and emerging health risks, particularly among Indigenous communities. The studies examined impacts from sea ice loss [14, 46], weather-related water contamination [36], human-made flooding [38], and health concerns among migrants [47].

Displacement and mobility challenges were prominent across these studies. Ballard et al. [38] described how the forced relocation of First Nations communities due to redirected flooding produced lasting mental, physical, and cultural harm. The study documented adaptation strategies that emphasized forgiveness, unity, cultural reclamation, land reconnection, and the nurturing of Minoaywin, an Indigenous concept of wellbeing that integrates physical, mental, and emotional balance.

Two studies focused specifically on Inuit communities’ adaptation to sea ice loss and seasonal changes that limit safe travel for cultural and subsistence activities [14, 46]. These works highlighted emotional and mental health effects, such as stress and anxiety, alongside physical risks. They also documented community-driven adaptive responses, including reinforcing cultural identity, deepening connections to the land, and expanding culturally relevant programs and research initiatives.

Other research addressed climate-linked health outcomes beyond Indigenous contexts. Harper et al. [36] investigated associations between precipitation changes and infectious gastrointestinal illness (IGI) in Inuit communities, recommending stronger water monitoring and treatment systems, improved community participation in environmental surveillance, and culturally appropriate dissemination of health information. Pinsk et al. [47] examined the elevated incidence of inflammatory bowel disease among South-East Asian migrant children, suggesting links to environmental changes following migration. Although climate-related exposures were not directly measured, the study underscored the need for targeted public health interventions and more comprehensive research on migrant health in Canada.

Taken together, these findings suggest that climate adaptation and mobility-related health responses must be culturally grounded, community-led, and supported by sustained infrastructure and policy investment. While Indigenous communities have developed adaptive practices rooted in traditional knowledge and cultural continuity, the literature reveals a notable lack of research examining climate-related mobility and health outcomes among non-Indigenous racialized populations in Canada.

### Conceptual framework application and comparative outcomes

Fourteen of the twenty-two studies explicitly used conceptual frameworks to guide their research. These included Indigenous epistemologies and justice-oriented approaches such as Two-Eyed Seeing [13, 42, 44, 48], the Anishinaabe Medicine Wheel [43], resurgence theory [34], Indigenous feminist perspectives [40], EcoHealth [49], and environmental justice or political ecology frameworks [14, 32, 37, 41, 45]. Studies employing these frameworks tended to situate climate-related health impacts within broader structural contexts, including colonialism, environmental governance, and systemic inequities. Framework-based studies consistently examined the structural determinants of health and explicitly linked climate impacts to systemic inequities, producing recommendations such as strengthening Indigenous data sovereignty [37, 49], implementing culturally grounded water monitoring [45], supporting food sovereignty programs [13, 32], and incorporating Elders in adaptation and governance structures [43, 46].

Framework studies often framed environmental change as both a biophysical hazard and a cultural threat, aligning adaptation with governance reform, self-determination, and cultural continuity. In contrast, the eight studies without explicit frameworks [25, 26, 33, 35, 38, 39, 47] focused more narrowly on exposure-outcome relationships, often providing limited discussion of systemic inequities or historical contexts. While valuable for quantifying health risks, these studies rarely addressed mobility, displacement, or racism, and tended to aggregate diverse racialized populations, which may obscure important social and health disparities.

Even among studies with frameworks, only a small subset explicitly incorporated climate justice, environmental racism, or mobility justice lenses [25, 32, 37, 40]. Overall, the comparative evidence suggests that justice-oriented conceptual frameworks strengthen the ability of climate-health research to generate policy-relevant and equity-focused recommendations. In their absence, research risks remaining descriptive rather than transformative, particularly for underrepresented racialized populations.

## Discussion

This scoping review provides an overview of the current state of Canadian scholarship examining the intersections of climate justice, mobility justice, and health inequities among racialized populations. The review identified 22 peer-reviewed studies spanning multiple disciplines and methodological approaches. Overall, the evidence base remains relatively limited but is gradually expanding, particularly in research involving Indigenous communities. Six thematic domains emerged from the synthesis: healthcare; food systems and security; environmental management and industrial development; housing, education, Indigenous governance and cultural preservation; emergency preparedness and disaster response; and climate adaptation and migration health. Together, these findings suggest that while climate-related health impacts among racialized populations are increasingly recognized in Canadian research, justice-oriented analytical frameworks and intersectional perspectives remain inconsistently applied.

Across the topics reviewed, healthcare emerged as a primary area of concern. Most studies focused on Indigenous populations in northern and remote communities, where slow-onset climate hazards such as ice thinning, unpredictable weather, and seasonal shifts were associated with both physical and mental health outcomes. Emotional, cultural, and spiritual health were emphasized, including concepts such as stress, solastalgia, and intergenerational trauma. These studies illustrate how health in Indigenous contexts is often understood as a multidimensional construct closely connected to land, culture, and historical relationships, with several authors noting discrepancies between Indigenous and Western approaches to measuring health outcomes [13, 34, 37, 39, 42, 43, 46, 48]. Importantly, the strong body of Indigenous-focused research provides foundational insights for understanding climate-related health inequities in Canada. However, comparable analyses remain limited for other racialized populations. This indicates an opportunity for future research to build on Indigenous scholarship while extending culturally grounded approaches to Black, immigrant, and other racialized communities. Notably, mental health outcomes were rarely discussed in the small subset of non-Indigenous-focused studies, suggesting that culturally responsive frameworks for understanding climate-related mental health among other racialized populations remain underdeveloped in Canadian scholarship.

Evidence from outside Canada shows that this gap can be addressed through the use of intersectional and culturally specific approaches. In the United States, for example, one study examined the mental health effects of Winter Storm Uri across multiple marginalized populations using an intersectional framework, finding that overlapping marginalized identities substantially amplified depression levels [50]. Other approaches have used targeted tools such as the Center for Epidemiologic Studies Depression Scale to compare outcomes between White and Black populations [51], or explored the sociology of place loss among Puerto Rican migrants post-Hurricane Maria, linking displacement and mobility constraints to mental wellbeing [52]. Similarly, Schwarzer and Schulz’s “Stressful Life Events” framework (2003) has been applied to assess post-traumatic stress disorder (PTSD) symptoms years after the 2010 Haiti earthquake, capturing the cumulative impact of multiple climatic and non-climatic stressors over time [53]. These examples illustrate how integrating intersectional and culturally specific frameworks can deepen understanding of climate-related health inequities among diverse populations.

Another notable limitation in the Canadian literature was the inconsistent application of conceptual frameworks to interpret structural inequities in climate-health outcomes. Only 14 of the 22 studies used such frameworks, with most rooted in Indigenous epistemologies such as Two-Eyed Seeing, the Anishinaabe Medicine Wheel, or community-developed models. Few explicitly applied justice-oriented approaches, including climate justice, environmental racism, or mobility justice, to analyze the unequal distribution of climate hazards and health vulnerabilities. This gap is notable given the extensive international literature documenting disproportionate environmental exposures among communities of colour resulting from historical segregation, policy neglect, and environmental racism [54–56]. The absence of such framing in Canada may perpetuate environmental inequities and limit the development of targeted climate-health responses.

In the United States, multiple studies and national assessments have documented that racialized populations are disproportionately affected by climate hazards such as extreme heat, flooding, and hurricanes. For instance, McKinsey and Company’s climate hazard analysis found that Black Americans are 1.4 times more likely to be exposed to extreme heat, 1.8 times more likely to experience hurricanes, and 1.6 times more likely to face a 1-in-100-year flood compared to the general population, with Black-owned homes in the Southeastern US facing disproportionately higher storm damage risk by 2050 [57]. These patterns mirror emerging Canadian evidence, such as the findings of Stieb et al. [25], which linked air pollution mortality to racialized urban geographies. The US experience underscores the value of race-based climate exposure data and its integration into equity-focused adaptation planning. Together, these findings highlight the importance of collecting and analyzing race-based environmental exposure data to inform equitable climate adaptation strategies.

Puerto Rico offers another instructive case. Following Hurricanes Irma and Maria, food insecurity and poor emergency response were compounded by structural racism and colonial neglect. Unlike the rapid mobilization seen in Texas and Florida after similar disasters, the federal response in Puerto Rico was delayed and under-resourced. Participants described being treated as “second-class citizens,” with the Jones Act restricting international shipments and exacerbating food shortages in a territory already reliant on imports for nearly half its food supply. In response, grassroots networks and the Puerto Rican diaspora mobilized community kitchens, supply distribution, and mutual aid [58]. These community-driven responses demonstrate how grassroots resilience strategies can emerge when institutional responses are insufficient and may offer insights for supporting marginalized communities facing climate-related crises in Canada.

Beyond the Indigenous context, a recurring problem in the reviewed Canadian literature was the aggregation of racialized groups into broad categories, obscuring health disparities within specific populations, particularly Black and immigrant communities. For example, while Stieb et al. [25] linked air pollution-related mortality to census tracts with high proportions of Black and Indigenous residents, they did not disaggregate the results by group. Similarly, Yu and Hu [26] examined the impacts of sociodemographic and environmental factors on COVID-19 but grouped all racialized individuals together at the neighborhood level. Such aggregation limits the ability to identify population-specific risks and design targeted climate-health interventions. By contrast, studies in the United States have stratified outcomes by racial group, including assessments of air pollution and COVID-19 spread [59], illustrating both the feasibility and necessity of disaggregated data. Applying similar approaches in Canada would strengthen climate-health research and improve the design of equitable adaptation strategies.

Although this study synthesized 22 peer-reviewed articles, none explicitly examined the links between mobility justice and health inequities. Yet, mobility injustice is a growing challenge for racialized and immigrant communities, many of whom lack access to safe, affordable, accessible, and reliable transportation. In 2019, almost one million residents across Canada’s eight largest cities were identified as being at risk of transport poverty, with racialized and immigrant populations disproportionately represented among them [60]. Transport poverty means not being able to access or afford adequate transportation. These inequities are further compounded by residential segregation, which often confines these groups to neighbourhoods with limited green space, bike lanes, and other essential amenities. Without reliable mobility options, marginalized and vulnerable populations face heightened risks of social and economic exclusion, with serious consequences for their health and well-being. Despite these realities, research examining the intersection of mobility injustice, climate vulnerability, and health among racialized populations in Canada remains limited, highlighting an important area for future research.

Taken together, the findings from this review reveal that while there is a growing body of Canadian research on climate and environmental health impacts, the literature remains overwhelmingly Indigenous-focused and underdeveloped for other racialized communities and equity deserving groups. Critical data gaps, particularly the lack of disaggregated and intersectional analyses, hinder the ability to design targeted and equitable interventions. Lessons from the United States, Puerto Rico, and other international contexts demonstrate that structural inequities, when left unaddressed, systematically intensify climate-related health risks for marginalized populations. Future research in Canada would benefit from prioritizing the systematic collection of race-based climate exposure data, incorporating justice-oriented theoretical frameworks, and co-developing climate adaptation strategies with affected communities. Without such measures, climate adaptation planning risks perpetuating the very inequities it seeks to resolve, leaving Black, immigrant, and other marginalized populations disproportionately exposed and insufficiently supported in the face of escalating climate crises.

### Strengths and limitations

A major strength of this scoping review is its comprehensive synthesis of peer-reviewed literature examining the intersections of climate change, environmental health, and racialized communities in Canada. The review used a rigorous and transparent search strategy reported in accordance with PRISMA-ScR guidelines to capture studies across multiple thematic areas, including healthcare, food systems, housing, governance, migration, and emergency preparedness. The inclusion of both quantitative and qualitative research, as well as studies grounded in conceptual and theoretical frameworks, allowed for a multifaceted understanding of structural and sociocultural determinants of health. By synthesizing evidence across diverse disciplinary fields, this review provides an integrated overview of how climate-related environmental changes interact with social inequities to influence health outcomes. The thematic analysis also identified critical representation gaps, particularly for non-Indigenous racialized communities, and applied an equity-focused perspective that can guide policy and practice development.

There are, however, important limitations. Restricting the review to peer-reviewed sources may have excluded relevant insights from grey literature, government reports, community-based research, and advocacy publications, which often reflect grassroots perspectives and emerging policy responses. The available literature is disproportionately centered on Indigenous communities, which limits the applicability of findings to other racialized groups such as Black, immigrant, and refugee populations in Canada. Additionally, many studies aggregated racialized populations into broad categories, limiting the ability to identify population-specific climate-health risks. Finally, the diversity of study designs, outcome measures, and conceptual approaches limited opportunities for direct comparison and prevented the use of meta-analytical techniques. However, such heterogeneity is typical in scoping reviews, which aim to map the breadth of evidence rather than statistically synthesize findings. These limitations highlight the urgent need for more inclusive, disaggregated, and justice-oriented climate-health research in the Canadian context.

### Policy implications

To address climate and environmental health inequities among racialized communities in Canada, the federal government should prioritize race-disaggregated and intersectional data collection to inform targeted interventions, while embedding environmental, climate, and mobility justice frameworks into adaptation and public health strategies. Improved data infrastructure would enable policymakers to better identify populations at heightened risk of climate-related health impacts and design evidence-informed responses. Federal and provincial funding bodies should create dedicated grant streams for community-led adaptation planning, with streamlined application processes for racialized community organizations. Municipal governments and public health agencies should strengthen emergency preparedness efforts in racialized communities through culturally appropriate mental health services, locally led response systems, and accessible communication in multiple languages.

Addressing structural vulnerabilities, including inadequate housing, environmental racism, and insecure employment, requires coordinated intersectoral policy action across all levels of government. Climate-health literacy should be enhanced by public health agencies and educational institutions through culturally tailored education initiatives in schools, workplaces, and community settings. Strengthening collaboration between policymakers, community organizations, and researchers will also be essential to ensure that climate adaptation strategies reflect the lived experiences and priorities of affected communities. Research funders and academic institutions should establish dedicated funding for research and interventions focusing on non-Indigenous racialized populations, with accountability and evaluation mechanisms embedded to measure progress and ensure the sustained reduction of inequities over time.

## Conclusion

This scoping review examined the intersections of climate justice, mobility justice, and health inequalities to map the existing literature and identify key gaps in knowledge within the Canadian context. Findings underscore the pressing need to integrate climate, environmental, and mobility justice principles into Canadian climate-health research, policy, and practice. Although most of the included studies center on Indigenous communities, there remains a substantial gap in evidence addressing the lived experiences of non-Indigenous racialized groups, particularly Black and immigrant populations.

The findings reveal that climate-related health inequities stem not only from environmental changes but also from entrenched colonial histories, systemic racism, and structural neglect, which manifest in inadequate housing, limited access to healthcare, food insecurity, and exclusion from governance and decision-making. Addressing these inequities therefore requires both environmental and social policy responses that confront structural determinants of health.

Addressing these inequities requires sustained commitment to collecting race-disaggregated and intersectional data, implementing culturally relevant adaptation strategies, and strengthening community-led governance. Future climate-health research in Canada should prioritize interdisciplinary collaboration and justice-oriented frameworks capable of examining how climate risks intersect with social inequities across diverse populations. Lessons from Indigenous and global equity-driven climate responses demonstrate that transformative, justice-focused action can protect health, wellbeing, and cultural continuity for all racialized communities facing the escalating impacts of climate change.

## Supplementary Information

Supplementary Material 1.

Supplementary Material 2.

Supplementary Material 3.

## Data Availability

All data generated or analyzed during this study are included in this published article and its supplementary materials. Additional details are available from the corresponding author upon reasonable request.

## Abbreviations

BARE: Black and Racial Equity Research Program
CIHR: Canadian Institutes of Health Research
COVID-19: Coronavirus Disease 2019
EcoHealth: Ecological Health Framework
IBD: Inflammatory Bowel Disease
IGI: Infectious Gastrointestinal Illness
MeHg: Methylmercury
NO₂: Nitrogen Dioxide
PM2.5: Particulate Matter ≤ 2.5 Micrometers
PRISMA-ScR: Preferred Reporting Items for Systematic Reviews and Meta-Analyses Extension for Scoping Reviews
PTSD: Post-Traumatic Stress Disorder
SAR: Search and Rescue
SDGs: Sustainable Development Goals
US: United States
WHO: World Health Organization
