# Supplementary material for: Climate justice, mobility justice, and health inequities among racialized communities in Canada: a scoping review
Source: Arch Public Health. 2026 Mar 29;84:105. doi: 10.1186/s13690-026-01906-2 (PMC13154671; doi:10.1186/s13690-026-01906-2)
Supplement: Supplementary file 2 — Supplementary Material 2. [file 13690_2026_1906_MOESM2_ESM.docx]

**Searches conducted by: Caitlin McClurg, MLIS. Libraries and Cultural Resources, University of Calgary.**

**Ovid MEDLINE(R) ALL <1946 to March 31, 2025>**

<https://ezproxy.lib.ucalgary.ca/login?url=https://ovidsp.ovid.com/ovidweb.cgi?T=JS&NEWS=N&PAGE=main&SHAREDSEARCHID=1bO4IqwM5jg46yR7Hqx7Kiun93Frdyh4ODSaFSwnKHoHpA4pxgK0cgV2mdVXBk92I>

1 Climate Change/ 33112

2 climate change.tw,kf. 78100

3 exp Natural Disasters/ 30373

4 natural disaster*.tw,kf. 6985

5 ((climat* or environment* or pollut*) adj3 (emergen* or disaster* or hazard* or crisis or impact* or implicat* or justice or injustice* or unjust or equit* or inequit* or disparit* or migrat* or displac* or relocat* or refugee* or forced or racis* or adversit*)).tw,kf. 89046

6 (extreme adj3 (weather or heat or temperature*)).tw,kf. 10005

7 high-temperature*.tw,kf. 70531

8 "high temperature*".tw,kf. 70531

9 "heat wave*".tw,kf. 2747

10 heat-wave*.tw,kf. 2747

11 "cold wave*".tw,kf. 184

12 cold-wave*.tw,kf. 184

13 hurricane*.tw,kf. 5019

14 wildfire*.tw,kf. 4713

15 drought*.tw,kf. 39652

16 flood*.tw,kf. 26640

17 tsunami*.tw,kf. 3120

18 monsoon*.tw,kf. 5837

19 avalanche*.tw,kf. 4346

20 landslide*.tw,kf. 1545

21 mudslide*.tw,kf. 76

22 sinkhole*.tw,kf. 239

23 cyclone*.tw,kf. 2705

24 tornado*.tw,kf. 936

25 haboob*.tw,kf. 9

26 "dust storm*".tw,kf. 846

27 sandstorm*.tw,kf. 232

28 earthquake*.tw,kf. 12093

29 "ice storm*".tw,kf. 140

30 thunderstorm*.tw,kf. 634

31 lightning.tw,kf. 2667

32 blizzard*.tw,kf. 247

33 volcan*.tw,kf. 10707

34 "weather event*".tw,kf. 2478

35 1 or 2 or 3 or 4 or 5 or 6 or 7 or 8 or 9 or 10 or 11 or 12 or 13 or 14 or 15 or 16 or 17 or 18 or 19 or 20 or 21 or 22 or 23 or 24 or 25 or 26 or 27 or 28 or 29 or 30 or 31 or 32 or 33 or 34 334645

36 exp Racial Groups/ 116711

37 exp "Black or African American"/ 77337

38 BIPOC.tw,kf. 497

39 ((Black or racial* or ethnic* or vulnerab* or displac* or migrat* or emigrat* or immigrat* or diaspor*) adj2 (adult* or population* or men or male* or women or female* or child* or youth* or adolescen* or individual* or minorit* or migrant* or refugee*)).tw,kf. 120699

40 36 or 37 or 38 or 39 217782

41 35 and 40 3745

42 exp "Systematic Review"/ 286150

43 systematic review.ti,pt. 355651

44 exp Meta-Analysis/ 216020

45 meta-analysis.ti,pt. 274135

46 meta analysis.ti,pt. 274135

47 review.ti,pt. 3808229

48 exp Congresses as Topic/ 32263

49 conference.ti,pt. 35166

50 42 or 43 or 44 or 45 or 46 or 47 or 48 or 49 3971024

51 41 not 50 3087

**Embase <1974 to 2025 March 31>**

<https://ezproxy.lib.ucalgary.ca/login?url=https://ovidsp.ovid.com/ovidweb.cgi?T=JS&NEWS=N&PAGE=main&SHAREDSEARCHID=3VrdZoTQijHngF784mXTEpAxZhnQCOn7AonH12s4vZdM4MLBBkZuMbwLiAoqsXk7k>

1 exp climate change/ 76869

2 climate change.tw,kf. 74659

3 exp natural disaster/ 5186

4 natural disaster*.tw,kf. 7751

5 ((climat* or environment* or pollut*) adj3 (emergen* or disaster* or hazard* or crisis or impact* or implicat* or justice or injustice* or unjust or equit* or inequit* or disparit* or migrat* or displac* or relocat* or refugee* or forced or racis* or adversit*)).tw,kf. 98092

6 (extreme adj3 (weather or heat or temperature*)).tw,kf. 9935

7 high-temperature*.tw,kf. 61041

8 "high temperature*".tw,kf. 61041

9 "heat wave*".tw,kf. 2906

10 heat-wave*.tw,kf. 2906

11 "cold wave*".tw,kf. 140

12 cold-wave*.tw,kf. 140

13 hurricane*.tw,kf. 5308

14 wildfire*.tw,kf. 4844

15 drought*.tw,kf. 34262

16 flood*.tw,kf. 28374

17 tsunami*.tw,kf. 3587

18 monsoon*.tw,kf. 6766

19 avalanche*.tw,kf. 3700

20 landslide*.tw,kf. 1431

21 mudslide*.tw,kf. 67

22 sinkhole*.tw,kf. 241

23 cyclone*.tw,kf. 3684

24 tornado*.tw,kf. 1046

25 haboob*.tw,kf. 5

26 "dust storm*".tw,kf. 1226

27 sandstorm*.tw,kf. 283

28 earthquake*.tw,kf. 12461

29 "ice storm*".tw,kf. 149

30 thunderstorm*.tw,kf. 729

31 lightning.tw,kf. 2627

32 blizzard*.tw,kf. 306

33 volcan*.tw,kf. 11111

34 "weather event*".tw,kf. 2452

35 1 or 2 or 3 or 4 or 5 or 6 or 7 or 8 or 9 or 10 or 11 or 12 or 13 or 14 or 15 or 16 or 17 or 18 or 19 or 20 or 21 or 22 or 23 or 24 or 25 or 26 or 27 or 28 or 29 or 30 or 31 or 32 or 33 or 34 349697

36 exp ethnic group/ 236983

37 exp Black person/ 182565

38 BIPOC.tw,kf. 631

39 ((Black or racial* or ethnic* or vulnerab* or displac* or migrat* or emigrat* or immigrat* or diaspor*) adj2 (adult* or population* or men or male* or women or female* or child* or youth* or adolescen* or individual* or minorit* or migrant* or refugee*)).tw,kf. 156943

40 36 or 37 or 38 or 39 513145

41 35 and 40 5345

42 exp "systematic review"/ 516731

43 systematic review.ti,pt. 322873

44 exp meta analysis/ 350683

45 meta-analysis.ti,pt. 263333

46 meta analysis.ti,pt. 263333

47 review.ti,pt. 3807792

48 exp conference abstract/ 2560338

49 exp conference paper/ 3378568

50 conference.ti,pt. 6236808

51 42 or 43 or 44 or 45 or 46 or 47 or 48 or 49 or 50 10101912

52 41 not 51 3719

53 limit 52 to human 3063

**APA PsycInfo <1806 to March 2025 Week 4>**

<https://ezproxy.lib.ucalgary.ca/login?url=https://ovidsp.ovid.com/ovidweb.cgi?T=JS&NEWS=N&PAGE=main&SHAREDSEARCHID=382QwoFVr1pS0FF7Y2Xdx1JKmcKaj34FI8Je1yFM1cQwjWK9CzS1LYL6A4tnaVAbQ>

1 Climate Change/ 4410

2 climate change.tw,id. 6378

3 exp Natural Disasters/ 6627

4 natural disaster*.tw,id. 4580

5 ((climat* or environment* or pollut*) adj3 (emergen* or disaster* or hazard* or crisis or impact* or implicat* or justice or injustice* or unjust or equit* or inequit* or disparit* or migrat* or displac* or relocat* or refugee* or forced or racis* or adversit*)).tw,id. 15528

6 (extreme adj3 (weather or heat or temperature*)).tw,id. 600

7 high-temperature*.tw,id. 542

8 "high temperature*".tw,id. 542

9 "heat wave*".tw,id. 155

10 heat-wave*.tw,id. 155

11 "cold wave*".tw,id. 6

12 cold-wave*.tw,id. 6

13 hurricane*.tw,id. 2805

14 wildfire*.tw,id. 423

15 drought*.tw,id. 611

16 flood*.tw,id. 3497

17 tsunami*.tw,id. 1073

18 monsoon*.tw,id. 70

19 avalanche*.tw,id. 312

20 landslide*.tw,id. 114

21 mudslide*.tw,id. 38

22 sinkhole*.tw,id. 12

23 cyclone*.tw,id. 147

24 tornado*.tw,id. 420

25 haboob*.tw,id. 0

26 "dust storm*".tw,id. 12

27 sandstorm*.tw,id. 11

28 earthquake*.tw,id. 3630

29 "ice storm*".tw,id. 46

30 thunderstorm*.tw,id. 85

31 lightning.tw,id. 401

32 blizzard*.tw,id. 71

33 volcan*.tw,id. 372

34 "weather event*".tw,id. 300

35 1 or 2 or 3 or 4 or 5 or 6 or 7 or 8 or 9 or 10 or 11 or 12 or 13 or 14 or 15 or 16 or 17 or 18 or 19 or 20 or 21 or 22 or 23 or 24 or 25 or 26 or 27 or 28 or 29 or 30 or 31 or 32 or 33 or 34 35746

36 exp Black People/ 65747

37 exp "Racial and Ethnic Groups"/ 182410

38 BIPOC.tw,id. 651

39 ((Black or racial* or ethnic* or vulnerab* or displac* or migrat* or emigrat* or immigrat* or diaspor*) adj2 (adult* or population* or men or male* or women or female* or child* or youth* or adolescen* or individual* or minorit* or migrant* or refugee*)).tw,id. 74700

40 36 or 37 or 38 or 39 228790

41 35 and 40 2434

42 exp "Systematic Review"/ 912

43 systematic review.ti,pt. 41936

44 exp Meta Analysis/ 5554

45 meta-analysis.ti,pt. 30004

46 meta analysis.ti,pt. 30004

47 review.ti,pt. 194901

48 conference.ti,pt. 3568

49 42 or 43 or 44 or 45 or 46 or 47 or 48 218081

50 41 not 49 2355

**Web of Science**

<https://www.webofscience.com/wos/woscc/summary/6f1446e4-56f7-48dd-a857-64d03bb0243e-0155a9b2c3/relevance/1>

Limit: Articles, 2000-2025, English/French

[Title] "climate change" OR environment* OR pollution OR "extreme weather" OR "extreme temperature*" OR "extreme heat" OR "high temperature*" OR "high-temperature*"

AND

[Abstract] refugee* OR Black OR migrant* OR racialized OR ethnic OR BIPOC

AND

[Title] racism OR injustice* OR justice OR unjust OR inequit* OR disparit* OR displac* OR forced or adversity

559

**Scopus**

Limits: Articles, 2000-2025, English/French

[Article title, Abstract, Keywords] "climate change" OR pollution OR "extreme weather" OR "extreme temperature*" OR "extreme heat" OR "high temperature*" OR "high-temperature*"

AND

[Article title, Abstract, Keywords] refugee* OR Black OR migrant* OR racialized OR ethnic OR BIPOC

AND

[Article title, Abstract, Keywords] racism OR injustice* OR justice OR unjust OR inequit* OR disparit* OR displac* OR forced or adversity

1855

**CINAHL**

Limits: 2002-2025, Academic Journals, English

[Abstract] "climate change" OR pollution OR "extreme weather" OR "extreme temperature*" OR "extreme heat" OR "high temperature*" OR "high-temperature*"

AND

[Abstract] refugee* OR Black OR migrant* OR racialized OR ethnic OR BIPOC

AND

[Abstract] racism OR injustice* OR justice OR unjust OR inequit* OR disparit* OR displac* OR forced or adversity

128

**Cochrane Central Register of Controlled Trials**

Limits: 2000-2025, English

[Title, Abstract, Keyword] climate NEXT change OR pollution OR extreme NEXT weather OR extreme NEXT temperature* OR extreme NEXT heat OR high NEXT temperature* OR high-temperature*

AND

[Title, Abstract, Keyword] refugee* OR Black OR migrant* OR racialized OR ethnic OR BIPOC

AND

[Title, Abstract, Keyword] racism OR injustice* OR justice OR unjust OR inequit* OR disparit* OR displac* OR forced or adversity

**77**Trials matching **climate NEXT change OR pollution OR extreme NEXT weather OR extreme NEXT temperature* OR extreme NEXT heat OR high NEXT temperature* OR high-temperature* in Title Abstract Keyword AND refugee* OR Black OR migrant* OR racialized OR ethnic OR BIPOC in Title Abstract Keyword AND racism OR injustice* OR justice OR unjust OR inequit* OR disparit* OR displac* OR forced or adversity in Title Abstract Keyword - (Word variations have been searched)**

**Environment Complete**

Limits: 2002-2025, Academic Journals, English/French

[Abstract or Author-Supplied Abstract] "climate change" OR pollution OR "extreme weather" OR "extreme temperature*" OR "extreme heat" OR "high temperature*" OR "high-temperature*"

AND

[Abstract or Author-Supplied Abstract] refugee* OR migrant* OR racialized OR ethnic OR BIPOC

AND

[Abstract or Author-Supplied Abstract] racism OR injustice* OR justice OR unjust OR inequit* OR disparit* OR displac* OR forced or adversity

229

**CAB Abstracts**

Limits: 2001-2025, Academic Journals, English

[Abstract] "climate change" OR pollution OR "extreme weather" OR "extreme temperature*" OR "extreme heat" OR "high temperature*" OR "high-temperature*"

AND

[Abstract] refugee* OR Black OR migrant* OR racialized OR ethnic OR BIPOC

AND

[Abstract] racism OR injustice* OR justice OR unjust OR inequit* OR disparit* OR displac* OR forced or adversity

140

**Social Work Abstracts**

[Abstract] "climate change" OR pollution OR "extreme weather" OR "extreme temperature*" OR "extreme heat" OR "high temperature*" OR "high-temperature*"

AND

[Abstract] refugee* OR Black OR migrant* OR racialized OR ethnic OR BIPOC

AND

[Abstract] racism OR injustice* OR justice OR unjust OR inequit* OR disparit* OR displac* OR forced or adversity

1

**SocINDEX with Full Text**

Limits: Academic Journals, 2000-2025, English

[Abstract or Author-Supplied Abstract] "climate change" OR pollution OR "extreme weather" OR "extreme temperature*" OR "extreme heat" OR "high temperature*" OR "high-temperature*"

AND

[Abstract or Author-Supplied Abstract] refugee* OR migrant* OR racialized OR ethnic OR Black OR BIPOC

AND

[Abstract or Author-Supplied Abstract] racism OR injustice* OR justice OR unjust OR inequit* OR disparit* OR displac* OR forced or adversity

133

**Policy Commons**

climate change environmental racism Black BIPOC refugees migrants

111

**Canada Commons**

climate change environmental racism Black BIPOC refugees migrants

42
